# Soil-transmitted helminthiasis among adolescents in Anaocha Local Government Area, Anambra State, Nigeria: Insights and recommendations for effective control

**DOI:** 10.1371/journal.pone.0292146

**Published:** 2025-01-07

**Authors:** Ogechukwu B. Aribodor, Eunice C. Jacob, Nwadiuto O. Azugo, Uche C. Ngenegbo, Ifeanyi Obika, Emmanuel M. Obikwelu, Obiageli J. Nebe

**Affiliations:** 1 Department of Zoology, Nnamdi Azikiwe University, Awka, Nigeria; 2 Social Innovation in Health Initiative (SIHI) Hub, Awka, Nigeria; 3 Department of Parasitology and Entomology, Nnamdi Azikiwe University, Awka, Nigeria; 4 Neglected Tropical Diseases Unit, Anambra State Ministry of Health, Awka, Nigeria; 5 Neglected Tropical Diseases Division, Federal Ministry of Health, Abuja, Nigeria; Hawassa University College of Medicine and Health Sciences, ETHIOPIA

## Abstract

Over the past decade, Anambra State, Nigeria, has implemented mass administration of medicines (MAMs) to combat soil-transmitted helminthiasis (STH), a significant public health challenge in low-income regions. Nevertheless, these efforts have predominantly focused on pre-school and school-aged children, leaving a notable gap in understanding STH infection rates and the efficacy of these campaigns among secondary school adolescents, who have been excluded from this initiative. Our study aimed to address this critical knowledge gap by assessing soil-transmitted helminthiasis (STH) prevalence and contextual factors hindering effective control among adolescents in Anambra State, Nigeria. We actively engaged 443 adolescents with a mean age of 14 years in a school-based cross-sectional study in selected communities within the Anaocha Local Government Area from 8 February to 7 July 2023 following informed consent and assent procedures. Employing a stratified random sampling technique, we collected demographic data and assessed STH risk factors using a structured questionnaire hosted on the Kobo Toolbox platform. For quantitative analysis of STH infections, the Kato-Katz technique was used. Analysis was performed using SPSS version 25, incorporating descriptive statistics and multinomial logistic regression, with statistical significance set at p<0.05. Of the 443 (213 males (48.0%) and 230 females (52.0%) adolescents studied, the overall prevalence of STH observed was 35.2% (156/443). *Ascaris lumbricoides* was the prevalent STH species (16.9%), followed by *Trichuris trichiura* (1.4%) and hookworm (0.5%). Only light-intensity infection was observed. Mixed infections were observed in 16.5% of adolescents, involving *A*. *lumbricoides* and hookworm (10.8%), followed by *A*. *lumbricoides* and *T*. *trichiura* (3.2%) and all three STH (2.5%). The observed overall prevalence was not statistically significant with respect to gender (OR: 0.961; 95% CI: 0.651–1.420; p > 0.05) or age (OR: 0.686; 95% CI: 0.459–1.025; p>0.05). Class (grade level) (OR = 1.75, 95% CI: 1.25–2.45, p = 0.003), knowledge and transmission of STH infection (OR = 0.60, 95% CI: 0.42–0.86, p = 0.008), parental occupation (OR = 1.90, 95% CI: 1.35–2.67, p < 0.001), parents’ literacy level (OR = 0.68, 95% CI: 0.48–0.96, p = 0.027), and the type of toilet (OR = 2.15, 95% CI: 1.54–3.00, p < 0.001) were all significantly correlated with STH infection. These findings highlight the role of adolescents in sustaining soil-transmitted helminthiasis (STH) transmission. Coupled with school-based deworming expansion, innovative improvements in water, sanitation, hygiene, and awareness can provide a cost-effective, sustainable solution for combatting STH infections in Anambra State.

## Introduction

Soil-transmitted helminthiasis (STH) remains a persistent global health challenge, imposing a disproportionate burden on impoverished communities with limited access to sanitation and clean water [1]. These neglected tropical diseases (NTDs) are predominantly driven by three major STH species: *Ascaris lumbricoides* (roundworm), *Trichuris trichiura* (whipworm), and the hookworms, *Ancylostoma duodenale* and *Necator americanus* [2]. Together, these parasitic infections account for approximately 85% of the global NTD burden, impacting nearly a quarter of the world’s population and resulting in 1.9 million years lived with disability (DALYs) [3, 4].

Globally, over 1.5 billion people are infected with soil-transmitted helminths, with approximately 800 million children at risk, particularly in tropical and subtropical regions of sub-Saharan Africa, the Americas, China, and East Asia [3]. This includes 265.3 million preschool-aged children (pre-SAC), 632.6 million school-aged children (SAC), 108 million adolescent girls, and 138.8 million pregnant and lactating women requiring regular deworming treatment to prevent STH [3].

Regions characterized by poor environmental sanitation, inadequate personal hygiene, limited educational opportunities, low socio-economic status, and constrained access to healthcare and safe drinking water consistently report higher STH prevalence rates [5]. Additionally, children born to mothers infected with these helminths during pregnancy are at elevated risk of infection during their early years [6]. Practices such as geophagy, open defecation, the use of leaves and paper for hygiene, nail-biting, and walking barefoot further contribute to the persistently high prevalence of STH infections [7–9].

Africa bears a significant burden of STH infections, with over 73 million preschool-age children and 160 million school-age children requiring treatment in 2022 [10]. Nigeria, endemic for all four STH species, faces the highest burden in sub-Saharan Africa, with prevalence rates exceeding 50% [12]. Despite progress towards the WHO 2020 NTD target of treating at least 75% of at-risk children, challenges persist due to regional disparities and overlooked demographic groups, particularly adolescents [11]. Several surveys revealed significant disparities of moderate-to-heavy intensity infections across all the states in the country. Notably, southern states grapple with higher prevalence rates compared to their northern counterparts, partly due to favourable environmental conditions such as high levels of precipitation and the low altitude of these areas [13].

The cornerstone of STH control lies in mass drug administration (MDA), complemented by health and hygiene education, and the provision of clean water and sanitary facilities to ensure the sustained impact of MDA. However, the effectiveness of these measures is hampered by the oversight of specific demographic groups, particularly adolescents, who often escape targeted interventions, leading to reinfection among treated children and others. In the African context, adolescents frequently assume caregiving roles in the absence of parents and play a pivotal role in shaping the health and well-being of younger individuals. Thus, their adoption of healthy practices and their role in educating others is of paramount significance.

In 2013, Anambra State initiated its state-wide NTD control program, distributing preventive chemotherapy (PC) to endemic areas with a primary focus on school-aged children in primary schools, typically aged 6–12 years. This focus inadvertently neglected older adolescents enrolled in secondary schools. Some academically advanced adolescents, who enroll in secondary schools at an earlier age, are eligible for the medication but become disenfranchised and missed during the Mass Drug Administration (MDA). While numerous studies have examined STH infections in Anambra State, a conspicuous gap remains, specifically regarding the burden of STH among adolescents [11–14]. Against the backdrop of a decade-long MDA initiative in Anambra State, it is imperative to furnish evidence-based insights into the STH status of adolescents, who represent the future workforce of both the state and Nigeria. Their health and well-being are intricately linked to the nation’s economic prosperity. Hence, this study aims to provide evidence-based insights into the endemicity of soil-transmitted helminthiasis among adolescents and identify barriers hindering effective control measures, crucial for informing targeted interventions and advancing public health efforts in the region.

## Materials and methods

### Study area

**Fig 1:**
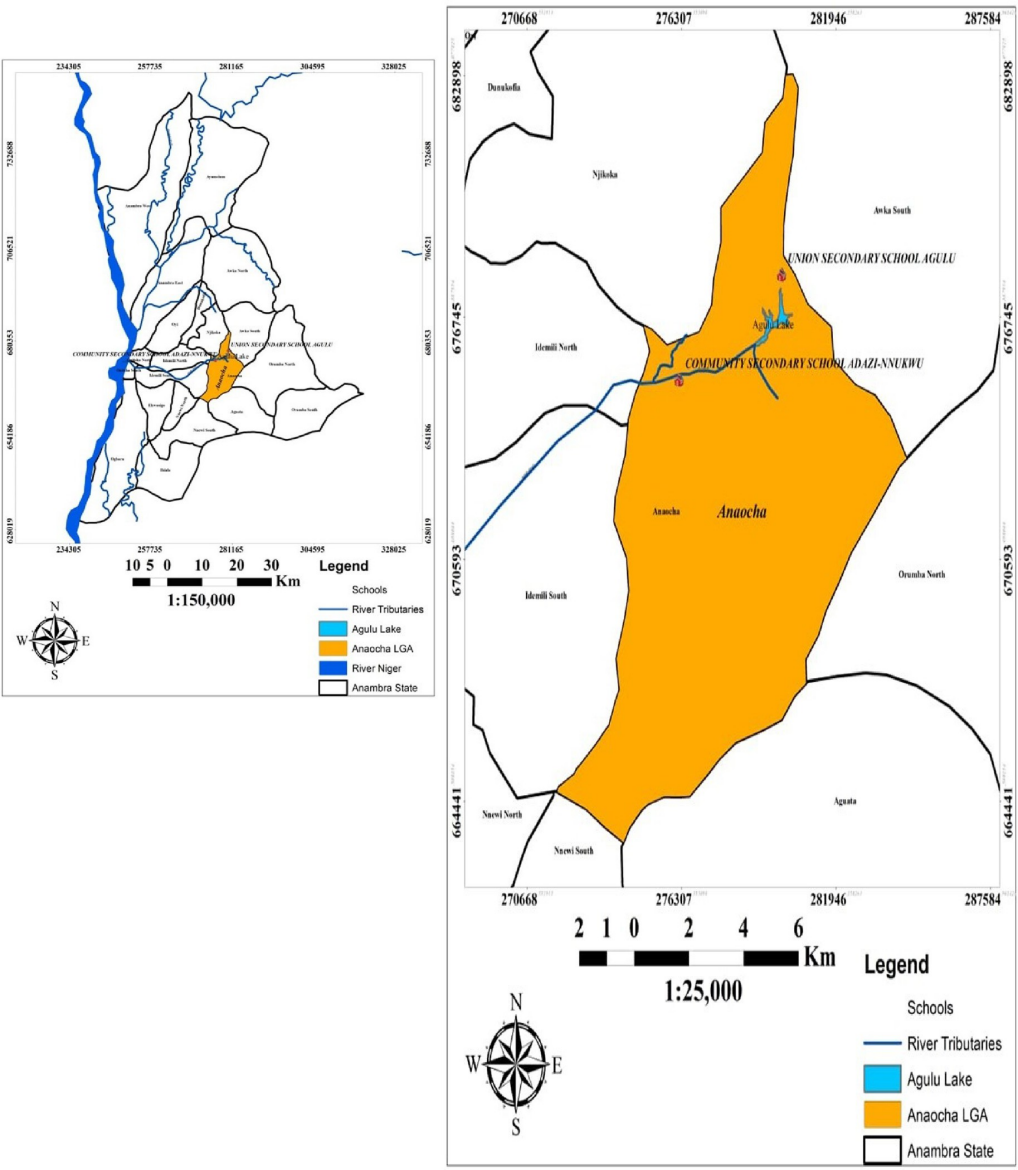
Map of the Anambra State showing the selected Local Government Area and the locations chosen for the study (source: Nnamdi Azikiwe University, geography information system laboratory, 2023) (the map was created using ArcGIS software using basemap: Https://Tile.Openstreetmap.Org/).

The study was carried out in the Anaocha Local Government Area (L.G.A.), situated in Anambra State, within the southeastern region of Nigeria (Fig 1). Anaocha L.G.A. is one of the twenty-one Local Government Areas in the state, spanning an area of approximately 171.62 square kilometres and hosting an estimated population of 418,360 distributed across ten communities [15, 16]. Two communities were randomly selected from the LGA namely, Adazi-Nnukwu and Agulu by ballot method to mitigate bias [17].

Adazi-Nnukwu, positioned at coordinates 6.1018° N and 7.0122° E within Anaocha LGA, is a community nestled within the Nri-Awka Area. In contrast, Agulu, located at coordinates 6.1160° N and 7.0678° E, serves as the largest town within Anaocha LGA. The populace is predominantly of the Igbo ethnic group who speak both Igbo and English languages. Christianity and traditional religions are the main religions practised in this area. The study area’s vegetation is classified under the tropical rainforest zone, though it has been significantly altered by various human activities, resulting in a remnant of trees and grasses. The region experiences a humid climate characterized by two distinct seasons: the dry season spanning from November to March and the rainy season from April to October. Topographic features in the study area encompass uplands, lowlands, stream channels, and gullies of variable dimensions. The elevation above sea level in both communities ranges from 65 metres to 212 metres. The average temperature is estimated at 25.5°C, and an annual rainfall of approximately 1,897 millimetres with relative humidity peaking at 85% during the rainy season and averaging 65% during the dry season. The majority of residents are farmers, traders, civil servants and teachers. The health centres and hospitals present in the L.G.A. grapple with critical challenges, chief among them being the shortage of qualified healthcare personnel and the absence of well-equipped laboratories. Also, there is a need for improved sanitary infrastructure as open defecation is a prevalent practice in these communities due to the lack of accessible public toilets. Additionally, it is pertinent to note that a significant majority of government-owned schools, commonly referred to as public schools, are often perceived as institutions catering primarily to economically disadvantaged students [13], further accentuating the complex issues surrounding access to quality education and healthcare within these areas.

### Study design

A school-based, cross-sectional study was conducted from 8 February to 7 July 2023. Only government-owned secondary schools that had a mixed population of male and female students were enrolled in the study. Eligible participants were adolescents aged 10 to 19 years who had resided in the chosen communities for at least three years and were enrolled in the selected secondary schools. Adolescents who were on medications or had taken anthelminthic drugs within a month before the commencement of the study were excluded from this study.

### Study population

The study population consisted of 470 consenting and assenting adolescents aged between 10–19 years who were enrolled in government-owned secondary schools namely: Union Secondary School, Agulu, and Community Secondary School, Adazi-Nnukwu.

### Sample size and sampling technique

The sample size of 443 was determined using the formula [18]: $n=\frac{N}{1+N{\left(e\right)}^{2}}$

Where:

n = sample size

N = population size

e = level of precision or sampling error which is taken to be 5% here.

Anambra State Population of Students as obtained from the Post Primary School Service Commission of Anambra State, N = 112, 255 [19]

e = 0.05

$$∴\;n=\frac{112255}{1+112255{\left(0.05\right)}^{2}}=398.6$$

To account for a 75% response rate [20] and a 25% non-response rate, an additional 11% of the sample size $\left(\frac{11}{100}\times 398.6=43.85+398.6=442.5\right)$ was calculated and included, resulting in a final sample size of 443 participants to optimize precision and minimize potential withdrawals. A stratified sampling technique was employed [20], with participants selected randomly from five strata representing different classes, ranging from junior secondary class 1 (JS 1) to senior secondary class 2 (SS2).

Inclusion criteria mandated that the participants be aged between 10 and 19, enrolled in one of the designated public secondary schools, and provided written consent for their involvement.

### Data collection

Data for this study were collected using a combination of qualitative and quantitative methods to ensure a comprehensive understanding of the research area and its participants. Qualitative data were gathered through structured questionnaires administered via one-on-one interviews conducted in English and Igbo, with rigorous translation back into English by fluent researchers. These questionnaires were designed to explore socio-demographics and various risk factors associated with soil-transmitted helminthiasis. Additionally, qualitative insights were enriched through focus group discussions (FGDs), where adolescents were grouped by gender and age into cohorts of five, providing deeper perspectives on the identified factors. Quantitative data, including demographic information and numerical risk factor assessments, were also collected using structured questionnaires with closed-ended questions. Data collection was facilitated using the KOBO ToolBox, an open-source tool for efficient data management and offline descriptive analysis [21], prioritizing privacy and gender sensitivity during questionnaire administration to respect cultural contexts. The complete dataset used for the risk factor assessment is available in (S1 File).

### Stool sample collection and examination

Stool samples were collected from each adolescent during school hours using labelled, sterilized, dry, leak-proof containers with screw caps. Each container was accurately labelled and coded to match the participant’s identity, and specimens were checked for proper labelling, quantity, and collection protocol. Collection during school hours ensured convenience, timely processing, and adherence to consistent sample collection procedures, guaranteeing the freshness and suitability of the specimens for subsequent analysis under supervised conditions. The stool samples were then stored in an ice-packed box and transported promptly to the Laboratory of the Department of Zoology, Nnamdi Azikiwe University, Awka, Anambra State, Nigeria within a maximum of an hour to prevent degradation and contamination. The standard Kato-Katz technique was used to examine stool specimens for the presence of *A*. *lumbricoides*, *T*. *trichiura*, and hookworm eggs. The prepared slides were examined under the microscope with ×10 and × 40 magnifications. All eggs detected in the preparations were identified, counted, and multiplied by a factor of 24, with the results represented as eggs per gram of stool (EPG) for the intensity of infection, according to WHO criteria [21]. All the Kato-Katz slides were prepared using 41.7 mg templates manufactured by Sterlitech Laboratories, United States of America, immediately after the stool specimens were received in the laboratory. Slide readings were conducted within the WHO-recommended timeframe of 30 to 60 minutes after preparation [22].

### Quality control/assurance

To ensure the quality of stool sample data, our study implemented rigorous quality control measures. Field assistants received training in stool sample collection techniques and adherence to precise protocols. We ensured that all collected samples were properly labelled and handled to maintain their integrity during transport to the laboratory. The completeness of the questionnaire and the quantity of the sample were checked daily.

In the laboratory, skilled technologists specialized in parasitology conducted initial examinations of all stool sample slides. As part of our quality assurance protocol, a random 10% subset of these slides underwent independent re-examination by another technologist. This double-checking process, performed with the technologist blinded to the initial results, aimed to verify egg counts accurately and minimize any potential for discrepancies and the risk of false positives. These measures were crucial in maintaining the accuracy and reliability of our stool sample data throughout the study.

### Data analysis

The collected data from the questionnaires were checked for completeness and consistency using Kobo toolbox [20]. The data was then exported in Excel format from Kobo toolbox to Microsoft Excel, where it was coded, and imported into the Statistical Package for the Social Sciences software version 25 (SPSS) for statistical analysis. Descriptive statistics such as frequencies and percentages were used to describe the demographic characteristics of the study participants. The mean intensity of the parasite EPG of stool was calculated for all infected individuals. Multinomial logistic regression analyses were employed to identify the association of each independent variable with the dependent variable. The strength of the association between risk factors and STH infection was tested by odds ratio with 95% confidence intervals. In all cases, a p-value less than 0.05 was considered statistically significant.

### Ethical approval and informed consent

Ethical clearance was obtained from the Health Research and Ethics Committee of Chukwuemeka Odumegwu Ojukwu University Teaching Hospital (COOUTH), Anambra (ref no: COOUTH/CMAC/ETH.C/Vol.1/FN:04/247) and an official permission was sought from Post Primary Schools Service Commission. The parents, guardians and teachers were sensitized during the Parents Teachers Association meetings in the selected schools on the aim and objectives of this research. Written informed consent was obtained from adolescents aged 18 to 19 years. Adolescents aged between 10 to 17 years gave assent to participate and completed assent forms through their parents or legal guardians. All infected adolescents enrolled for this study were treated with Albendazole.

## Results

### Socio-demographic, socio-economic characteristics and hygiene conditions of the adolescents studied

As presented in Table 1, a total of 443 adolescents aged 10–19 years old were recruited for this study from the schools visited. Among them, 51.9% (230/443) were females and 48.1% (213/443) were males. The mean age of the study participants was 14.09 years (SD: ±2.47 years). Adolescents between the ages of 10–14 years were 58.2% (258/443), while 41.8% (185/443) were between the ages of 15–19 years. Also, 61.4% (272/443) and 38.6 (171/443) of the adolescents studied were from Agulu and Adazi-Nnukwu respectively. Most of the study participants (65.9%, 292/443) had parents who were traders/entrepreneurs. A majority of them had parents who had completed secondary school education, 63.7% (282/443). Toilet facilities were present in both schools; however, they were restricted to staff use only, depriving students of essential facilities.

**Table 1:** Socio-demographic, socio-economic characteristics and hygiene conditions of adolescents studied in Anaocha L.G.A., Anambra State, Nigeria (N = 443).

Variable	Category	Frequency (n)	Percentage (%)
Gender	Male	213	48.1
	Female	230	51.9
Age (years)	10–14	258	58.2
	15–19	185	41.8
Location/School	Community Secondary School, Adazi-Nnukwu	171	38.6
	Union Secondary School, Agulu	272	61.4
Parents’ occupation	Traders/Entrepreneurs	292	65.9
	Teachers/Civil Servants	57	12.9
	Farmers	64	14.4
	Drivers	8	1.9
	Professionals (lawyers and health practitioners)	22	4.9
Parents’ literacy level	No formal education	13	2.9
	Primary education	72	16.3
	Secondary education	282	63.7
	Tertiary education	76	17.2
Water facility in the school	Not available	272	61.4
	Available	171	38.6
Water facility in the home	Not available	21	4.7
	Poor	27	6.1
	Fair	113	25.5
	Good	199	44.9
	Excellent	83	18.7
Drinking water source	Borehole/tap	365	82.4
	Sachet/bottled water	51	11.5
	River/Stream	27	6.1
Hand washing facility in school	Available	171	38.6
	Not available	272	61.4
Hand washing facility at home	Not available	28	6.3
	Poor	63	14.2
	Fair	135	30.5
	Good	173	39.1
	Excellent	44	9.9
Toilet in the home	Available	418	94.4
	Not available	25	5.6
Type of toilet in the home	Water cistern	292	65.9
	Pit toilet	126	28.4
	Bush/open defecation	25	5.6
Toilet in the school	Accessible	0	0.0
	Not accessible	443	100.0

### The overall prevalence of soil-transmitted helminths infection among adolescents studied

The overall prevalence of STH infection among adolescents studied was found to be 35.2% (156/443) (95% CI: 30.6–39.7). Parasite-specific infection prevalence was 16.9% (75/443) for *A*. *lumbricoides*, 1.4% (6/443) for *T*. *trichiura*, and 0.5% (2/443) for hookworm. Among the adolescents studied, 18.7% (83/443) had a single infection, while 16.5% (73/443) had multiple infections. The most prevalent co-infections were *A*. *lumbricoides* and hookworm, occurring in 10.8% (48/443) of the cases, followed by *A*. *lumbricoides* and *T*. *trichiura* in 3.2% (14/443) of the cases, and a combination of *A*. *lumbricoides*, *T*. *trichiura*, and hookworm in 2.5% (11/443) of the cases. The overall prevalence of STH infection was slightly higher in males 35.7% (76/213) than in females 34.8% (80/230) (OR: 0.961; 95% CI: 0.651–1.420; p = 0.843). By age group, adolescents aged 10–14 years had a higher prevalence (38.8%, 100/258) compared to those aged 15–19 years (30.3%, 56/185), though this difference was not statistically significant (OR: 0.686, 95% CI: 0.459–1.025, p = 0.066).

Regarding location, adolescents from Adazi-Nnukwu had a higher prevalence (40.4%, 69/171) compared to those from Agulu (32.0%, 87/272), with an odds ratio of 0.695 (95% CI: 0.467–1.035, p = 0.073). (Table 2).

**Table 2:** The overall prevalence of soil-transmitted helminths among adolescents studied in Anaocha L.G.A., Anambra State, Nigeria.

(Total NE = 443) Gender		Mono-infection			Co-infection		Triple Infection			
	NE	*Ascaris lumbricoides* NI (%)	Hookworm NI (%)	*T*. *trichiura* NI (%)	*A*. *lumbricoides* and *T*. *trichiura* NI (%)	*A*. *lumbricoides* and Hookworm NI (%)	*A*. *lumbricoides*, *T*. *trichiura* and Hookworm NI (%)	Total NI (%)	OR (95% CI)	P-value
Male	213	32 (15.0)	2 (0.9)	6 (2.8)	5 (2.3)	25 (11.7)	6 (2.8)	76 (35.7)	0.961 (0.651–1.420)	0.843^NS^
Female^*^	230	43 (18.7)	0 (0.0)	0 (0.0)	9 (3.9)	23 (10.0)	5 (2.2)	80 (34.8)	1.00	
**Age (years)**										
10–14	258	33 (12.8)	2 (0.8)	3 (1.2)	11 (4.3)	40 (15.5)	11 (4.3)	100 (38.8)	0.686 (0.459–1.025)	0.066^NS^
15–19^*^	185	42 (22.7)	0 (0.0)	3 (1.6)	3 (1.6)	8 (4.3)	0 (0.0)	56 (30.3)	1.00	
**Location**										
Adazi-Nnukwu	171	45(26.3)	0 (0.0)	6 (3.5)	6 (3.5)	9 (5.3)	3(1.8)	69(40.4)	0.695 (0.467–1.035)	0.073^NS^
Agulu^*^	272	30(11.0)	2 (0.7)	0 (0.0)	8 (2.9)	39(14.3)	8(2.9)	87(32.0)	1.00	

OR = Odds ratio, CI = Confidence interval, ^*^ = Reference category, ^S^ = significant (p<0.05), ^NS^ = Not significant (p>0.05), NE = Number Examined. NI = Number Infected.

### The intensity of soil-transmitted helminths infection among adolescents studied

Results showed that all 156 adolescents with STH infections had light-intensity infections of *A*. *lumbricoides*, hookworm, and *T*. *trichiura*. The overall mean EPG of *A*. *lumbricoides* was 109.14 (95% CI: 95.77–122.50). However, female adolescents had a higher arithmetic mean EPG of 109.4 (95% CI: 92.14–126.66) than males, whose mean EPG was 108.85 (95% CI: 87.77–129.92). Meanwhile, male adolescents infected with *T*. *trichiura* had a higher mean EPG of 39.43 (95% CI: 27.76–51.09) compared to females whose mean EPG was 32.47 (95% CI: 26.39–38.55). The overall mean EPG for *T*. *trichiura* was 35.61 (95% CI: 29.65–41.57), and for hookworm, it was 59.02 (95% CI: 32.05–85.99) (Table 3).

**Table 3:** The intensity of soil-transmitted helminths infection among adolescents studied in Anaocha L.G.A., Anambra State, Nigeria.

STH	Category	Number Infected (%)	Mean intensity ($\bar{\text{x}}$ ± σ)	EPG Range	95% Confidence Interval
***A*. *lumbricoides***	**Gender**				
	Male	71 (48.0)	108.85 ± 10.76	(24-360)	(87.77-129.92)
	Female	77 (52.0)	109.40 ± 8.84	(24-360)	(92.14-126.66)
	**Age (years)**				
	10-14	98 (66.2)	104.33 ± 7.83	(24-360)	(89.0-119.65)
	15-19	50 (33.8)	118.56 ± 13.49	(24-360)	(92.22-144.9)
	**Community**				
	Adazi-Nnukwu	63 (42.6)	116.57 ± 11.36	(24-360)	(94.38-138.76)
	Agulu	85 (57.4)	103.62 ± 8.54	(24-360)	(86.86-120.38)
	**Overall**	**148 (100)**	**109.14 ± 6.83**	**(24-360)**	**(95.77-122.50)**
***T*. *trichiura***	**Gender**				
	Male	14 (45.2)	39.43 ± 5.95	(24-72)	(27.76-51.09)
	Female	17 (54.8)	32.47 ± 3.11	(24-48)	(26.39-38.55)
	**Age (years)**				
	10-14	22 (71.0)	30.55± 2.48	(24-48)	(25.69-35.40)
	15-19	9 (29.0)	48.0± 8.19	(24-72)	(32.02-63.98)
	**Community**				
	Adazi-Nnukwu	15 (48.4)	38.4 ± 5.61	(24-72)	(27.39-49.41)
	Agulu	16 (51.6)	33.0 ± 3.26	(24-48)	(26.61-39.39)
	**Overall**	**31 (100)**	**35.61 ± 3.04**	**(24-72)**	**(29.65-41.57)**
**Hookworm**	**Gender**				
	Male	33 (54.1)	47.27± 5.91	(24-120)	(35.72-58.83)
	Female	28 (45.9)	72.86 ± 30.05	(24-840)	(14.03-131.69)
	**Age (years)**				
	10-14	53 (86.9)	60.68 ± 15.84	(24-840)	(29.61-91.75)
	15-19	8 (13.1)	48.0 ± 9.49	(24-72)	(29.42-66.58)
	**Community**				
	Adazi Nnukwu	12 (19.7)	36.0 ± 4.06	(24-48)	(28.04-43.96)
	Agulu	49 (80.3)	64.65 ± 17.13	(24-840)	(31.08-98.22)
	**Overall**	**61 (100)**	**59.02 ± 13.99**	**(24-840)**	**(32.05-85.99)**

### Risk factors of soil-transmitted helminths infections among adolescents studied

The results showed that adolescents who did not know about STH infection (OR: 4.286; 95% CI: 1.423–12.906; p<0.05) were significantly 4.3.times more likely to be infected with one or more species of STH than those who had prior knowledge (Table 4). Furthermore, adolescents who received education about STH infections in school (OR: 0.455; 95% CI: 0.276–0.749; p<0.05) and through MAMs (OR: 0.224; 95% CI: 0.108–0.461; p<0.05) were less likely to be infected compared to those who did not know about the infection (Table 4).

**Table 4:** Multinomial logistic regression analysis of risk factors associated with any soil-transmitted helminths infection among adolescents studied in Anaocha L.G.A., Anambra State (n = 443).

	Category	NE	NI (%)	OR (95% CI)	p-value
**STH knowledge**	No knowledge	135	32 (23.7)	4.286 (1.423–12.906)	0.010^S^
	Slightly knowledgeable	293	115 (38.2)	2.424 (0.840–6.994)	0.101^NS^
	Very knowledgeable*	15	9 (60.0)	1.00	
**Source of knowledge**	School	175	71 (40.6)	0.455 (0.276–0.749)	0.002^S^
	Mass media	53	18 (34.0)	0.604 (0.302–1.208)	0.154^NS^
	Mass administration of medicines (MAMs)	80	35 (43.8)	0.399 (0.221–0.723)	0.002^S^
	No*	135	32 (23.7)	1.00	
**STH transmission**	Skin penetration	5	2 (40.0)	0.066 (0.015–0.284)	0.001^S^
	No idea	360	122 (33.9)	5.526 (3.298–9.270)	0.001^S^
	Oral-faecal route	11	2 (18.6)	3.650 (0.732–18.181)	0.114^NS^
	Food and water-borne route^*^	67	30 (44.8)	1.00	
**Parents’ occupation**	Traders/Entrepreneurs	292	90 (30.8)	4.810 (1.896–12.201)	0.001^S^
	Teachers/Civil Servants	57	21 (36.8)	3.673 (1.290–10.458)	0.015^S^
	Farmers	64	30 (46.9)	2.429 (0.873–6.753)	0.089^NS^
	Drivers	8	0 (0.0)	X	
	Professionals (lawyers/health practitioners)	22	15 (68.2)	1.00	
**Parents’ Literacy Level**	No formal education	13	6 (46.2)	0.996 (0.306–3.241)	0.995^NS^
	Primary education	72	18 (25.0)	2.561 (1.274–5.150)	0.008^S^
	Secondary education	282	97 (34.4)	1.628 (0.974–2.721)	0.063^NS^
	Tertiary education*	76	35 (46.1)	1.00	
**Class**	JSS 1	171	86 (50.3)	0.291 (0.152–0.556)	0.000^S^
	JSS 2	91	14 (15.4)	1.618 (0.720–3.635)	0.244^NS^
	JSS 3	45	21 (46.7)	0.336 (0.148–0.764)	0.009^S^
	SSS 1	70	20 (28.6)	0.735 (0.339–1.596)	0.437^NS^
	SSS 2^*^	66	15 (22.7)	1.00	
**Water facility in school**	Not available	272	87 (32.0)	1.438 (0.966–2.141)	0.073^NS^
	Available*	171	69 (40.4)	1.00	
**Water facility in the home**	Not available	21	4 (19.0)	3.099 (0.959–10.016)	0.059^NS^
	Poor	27	7 (25.9)	2.083 (0.794–5.467)	0.136^NS^
	Fair	113	46 (40.7)	1.062 (0.598–1.887)	0.873^NS^
	Good	199	64 (32.2)	1.538 (0.908–2.607)	0.110^NS^
	Excellent^*^	83	35 (42.2)	1.00	
**Drinking water source**	Borehole/tap	365	133 (36.4)	0.498 (0.196–1.266)	0.143^NS^
	Sachet/bottled water	51	17 (33.3)	0.571 (0.194–1.679)	0.309^NS^
	River/Stream^*^	27	6 (22.2)	1.00	
**Toilet type at home**	Water cistern	292	105(36.0)	2.671 (1.159–6.158)	0.021^S^
	Pit toilet	126	36 (28.6)	3.750 (1.542–9.119)	0.004^S^
	Bush/open defecation^*^	25	15 (60.0)	1.00	
**Hand washing facility in school**	Not available	272	87 (32.0)	1.438 (0.966–2.141)	0.073^NS^
	Available^*^	171	69 (40.4)	1.00	
**Hand washing facility at home**	Poor	63	23 (36.5)	0.966 (0.382–2.443)	0.942^NS^
	Fair	135	45 (33.3)	1.111 (0.474–2.604)	0.808^NS^
	Good	173	66 (38.2)	0.901 (0.392–2.069)	0.805^NS^
	Excellent	44	12 (27.3)	1.481 (0.535–4.103)	0.450^NS^
	Not available^*^	28	10 (35.7)	1.00	
**Hand washing after defecation**	Always	0	0	X	
	Often	134	35 (26.1)	1.665 (1.015–2.733)	0.044^S^
	Sometimes	139	58 (41.7)	0.822 (0.520–1.301)	0.403^NS^
	Never^*^	170	63 (37.1)	1.00	
**Washing fruits**	Always	0	0	X	
	Often	66	27 (40.9)	0.807 (0.465–1.401)	0.446^NS^
	Sometimes	112	34 (30.4)	1.286 (0.798–2.061)	0.305^NS^
	Never^*^	265	95 (35.8)	1.00	
**Hand washing before and after meals**	Always	59	21 (35.6)	0.995 (0.540–1.835)	0.988^NS^
	Often	198	69 (34.8)	1.028 (0.676–1.564)	0.896^NS^
	Never	0	0	X	
	Sometimes^*^	186	66 (35.5)	1.00	
**Nail hygiene**	Well-trimmed and dirty	177	75 (42.2)	0.503 (0.319–0.794)	0.003^S^
	Untrimmed and dirty	62	34 (54.8)	0.304 (0.166–0.559)	0.000^S^
	Untrimmed and clean	41	3 (7.3)	4.683 (1.376–15.947)	0.014^S^
	Well-trimmed and clean^*^	163	44 (27.0)	1.00	
**Nail biting**	Yes	133	50 (37.6)	0.789 (0.500–1.244)	0.308^NS^
	Sometimes	102	39 (38.2)	0.768 (0.468–1.258)	0.294^NS^
	No^*^	208	67 (32.2)	1.00	
**Walking barefoot**	Yes	110	43 (39.1)	0.563 (0.331–0.956)	0.033^S^
	Sometimes	186	74 (39.8)	0.547 (0.342–0.874)	0.012^S^
	No*	147	39 (26.5)	1.00	
**STH treatment**	Yes	289	108 (37.4)	0.759 (0.501–1.150)	0.194^NS^
	No^*^	154	48 (31.2)	1.00	
**Source of STH treatment**	Mass administration of medicines (MAMs)	82	34 (41.5)	0.639 (0.367–1.115)	0.115^NS^
	Over the counter	183	62 (33.9)	0.884 (0.559–1.397)	0.597^NS^
	Hospitals	24	12 (50.0)	0.453 (0.190–1.081)	0.074^NS^
	No^*^	154	48 (31.2)	1.00	
**Duration of last deworming**	1–3 months	37	22 (59.5)	0.309 (0.147–0.647)	0.002^S^
	4–6 months	35	15 (42.9)	0.604 (0.285–1.280)	0.188^NS^
	Above 6 months	217	71 (32.7)	0.931 (0.598–1.451)	0.753^NS^
	No^*^	154	48 (31.2)	1.00	
**Who administered the medicine?**	Family member	165	64 (38.8)	0.715 (0.450–1.135)	0.155^NS^
	Health practitioner	124	44 (35.5)	0.823 (0.499–1.360)	0.447^NS^
	No^*^	154	48 (31.2)	1.00	
**How important do you think it is to prevent STH infections?**	Very important	356	119 (33.4)	1.328 (0.219–8.054)	0.758^NS^
	Somewhat important	82	35 (42.7)	0.895 (0.142–5.648)	0.906^NS^
	Not important^*^	5	2 (40.0)	1.00	

NE = Number examined, NI = Number Infected, OR = Odds ratio, CI = Confidence interval, ^*^ = Reference category, ^S^ = significant (p<0.05), ^NS^ = Not significant (p>0.05)

**Fig 2:**
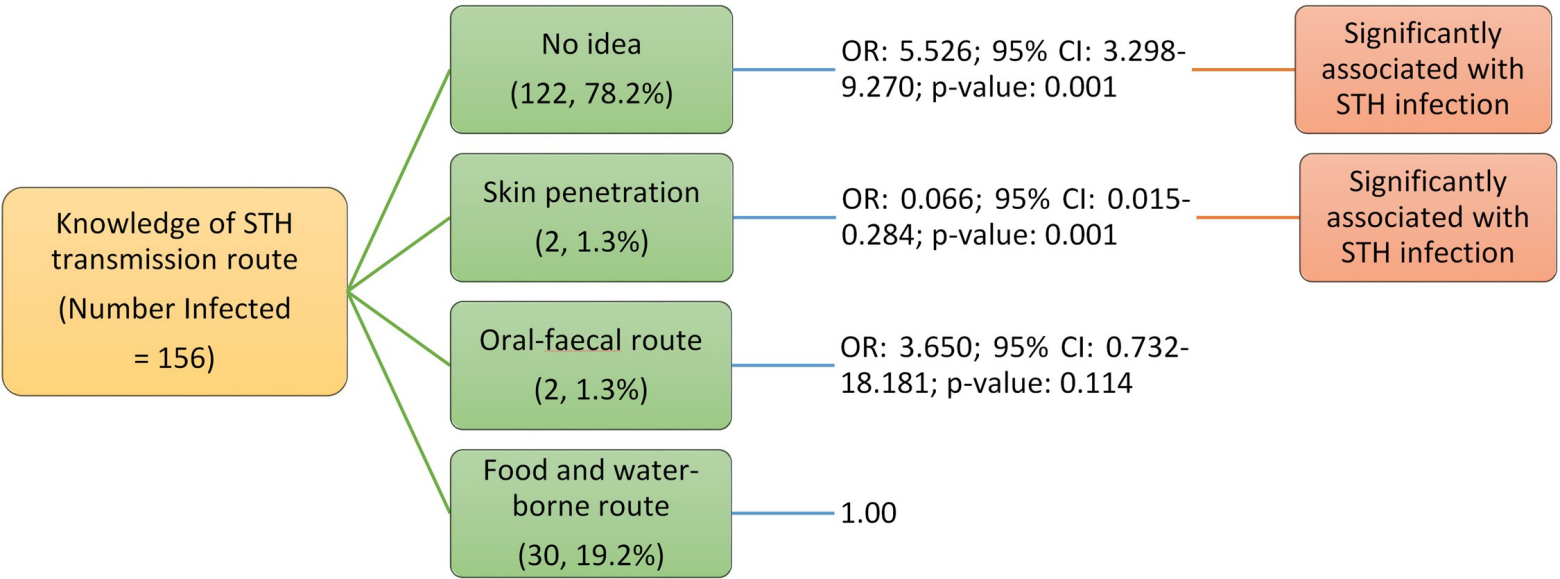
Association of knowledge of infection transmission route with soil-transmitted helminths infection among adolescents studied in Anaocha L.G.A., Anambra State.

Adolescents who had no idea how STH infection was transmitted (OR: 5.526; 95% CI: 3.298–9.270; p<0.05) were 5.5 times more likely to be infected (Fig 2) (Table 4).

**Fig 3:**
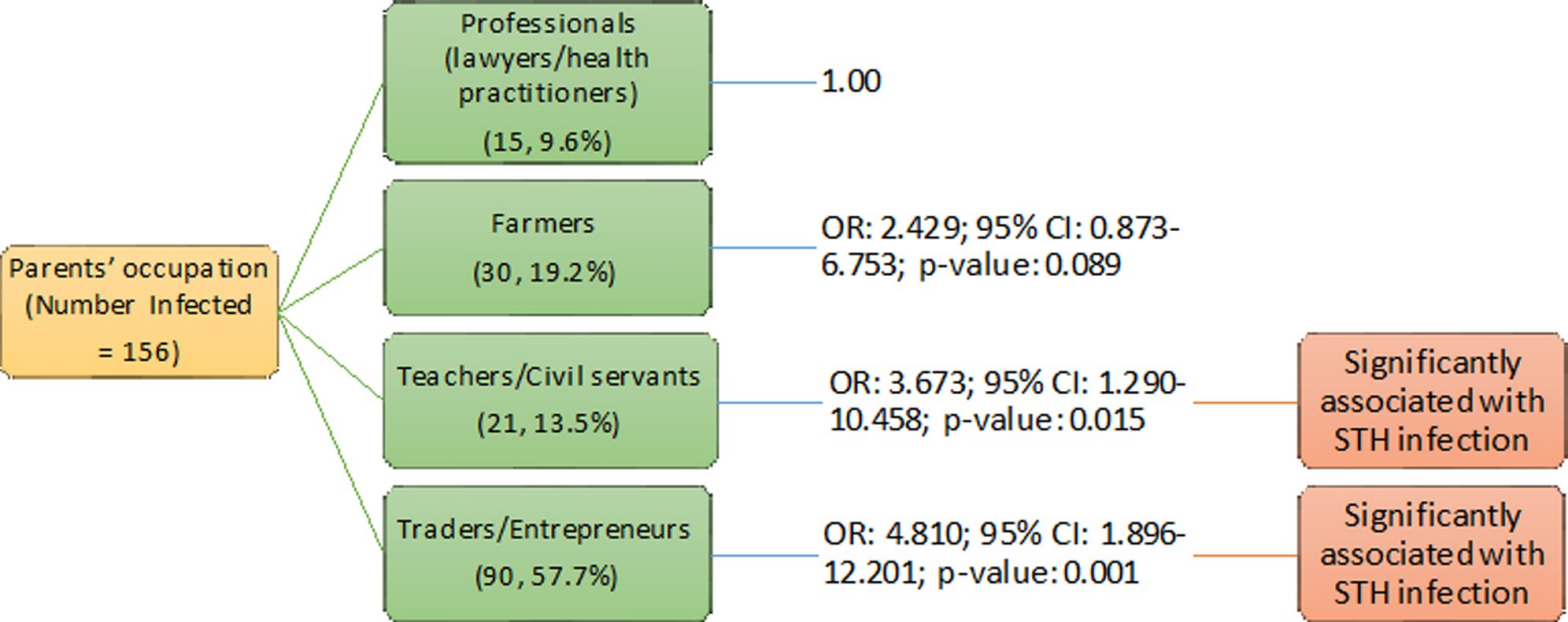
Association of parents’ occupation with soil-transmitted helminths infection among adolescents studied in Anaocha L.G.A., Anambra State.

**Fig 4:**
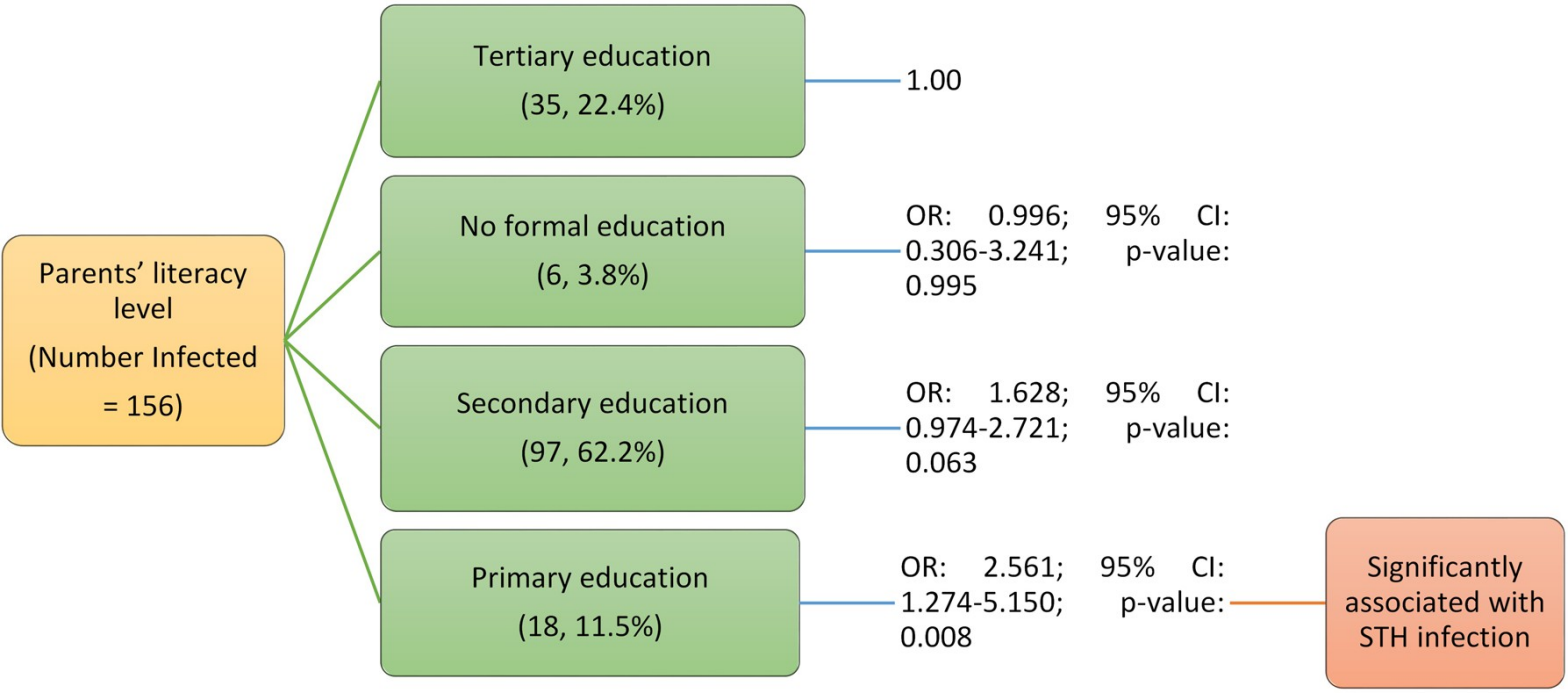
Association of parents’ literacy level with soil-transmitted helminths infection among adolescents studied in Anaocha L.G.A., Anambra State.

Regarding STH infection with parents’ occupation indicates that adolescents whose parents were traders/entrepreneurs (OR: 7.856; 95% CI: 1.600–38.559; p<0.05) and teacher/civil servants (OR: 6.000; 95% CI: 1.140–31.590; p<0.05) were significantly more likely to be infected (Fig 3) (Table 4). Another significant finding was the level of education of their parents, those whose parents completed only primary education (OR: 2.561; 95% CI: 1.274–5.150; p<0.05) were 2.6 times more likely to be infected with STH infection compared to those whose parents attained tertiary education (Fig 4) (Table 4).

Adolescents in JSS 1 class (OR: 0.291; 95% CI: 0.152–0.556; p<0.05) and JSS 3 class (OR: 0.336; 95% CI: 0.148–0.764; p<0.05) were significantly less likely to be infected compared to adolescents in SSS 2 class (Table 4). Adolescents who used pit toilets (OR: 3.750; 95% CI: 1.542–9.119; p<0.05) and water cisterns (OR: 2.671; 95% CI: 1.159–6.158; p<0.05) were more likely to be infected than those who practiced open defecation (Table 4).

Gender, age, source of drinking water, biting nails, washing hands before and after meals, and washing fruits did not have any significant statistical relationship with positive diagnosis of STH infection.

## Discussion

The prevalence of soil-transmitted helminthiasis among adolescents in Anaocha LGA was found to be 35.2%, classifying the area as a "moderate risk area" for preventive therapy according to WHO standards [23, 24]. This highlights the endemic nature of STH infections in Adazi-Nnukwu and Agulu despite mass drug administration efforts since 2013. The persistent prevalence among adolescents, who are often overlooked by current school-based programs targeting younger children, poses a significant challenge to disease eradication efforts. This is because infected adolescents may act as significant carriers of this infection, contributing to its continuous transmission within the community.

*Ascaris lumbricoides* emerged as the predominant parasite, followed by *Trichuris trichiura* and hookworm, reflecting earlier studies [11, 12, 14]. The resilience of *A*. *lumbricoides* eggs in harsh environments could contribute to its dominance. The study also revealed a notable 16.5% co-infection rate, predominantly with *A*. *lumbricoides* and hookworm. These co-infections significantly heighten the risk of severe morbidity associated with helminth infections. Adolescent girls are especially vulnerable to compounded health issues such as severe anemia, malnutrition, and impaired cognitive and physical development, which can negatively impact their reproductive health and future opportunities.

Key socioeconomic factors such as parental occupation and education significantly influence infection rates, with traders and entrepreneurs’ children more vulnerable. Poor hygiene practices and inadequate sanitation facilities in schools and homes further exacerbate the risk. The lack of access to safe and functional toilets in schools due to the lack of a steady water supply forces students to resort to open defecation, increasing their exposure and that of other community members to STH infections. This concurs with findings from similar studies conducted in Nigeria [12, 14, 25, 26].

The lack of access to sanitation facilities in schools, where toilets were restricted to staff use only, forces students to resort to open defecation and urination in surrounding bushes, posing significant implications for their health, safety, and dignity. This has far-reaching implications for global health, as inadequate sanitation can contaminate water sources and increase the risk of waterborne diseases. This risk is particularly heightened during flooding events in areas like Anambra State, where floodwaters can spread faecal matter and other contaminants, increasing the prevalence of diseases such as cholera, typhoid, and dysentery. Therefore, addressing sanitation infrastructure and ensuring the availability and accessibility of toilets and a steady water supply is crucial to mitigate these public health risks and ensure the well-being of students and the entire community.

Notably, adolescents dewormed within 1–3 months before the study showed significantly lower infection rates, underscoring the importance of regular, timely treatment. However, the high reinfection rate post-treatment highlights ongoing environmental and behavioural challenges in these communities. These findings are in agreement with previous works [11, 27–30] which adduced that without improvement in hygiene and access to safe water, other interventions are rendered ineffective.

The study recommends revising treatment strategies to comprehensively include adolescents in mass drug administration programs. Innovative interventions such as health education through creative mediums (theatre, art, music) and enhanced sanitation infrastructure are crucial. Policies should prioritize holistic approaches that address environmental, socioeconomic, and behavioural factors to effectively curb STH transmission.

The role of religious leaders in health education emerged as pivotal in community engagement regarding STH prevention and treatment. Despite churches primarily focusing on spiritual matters, they play a significant role in disseminating health information. Participants reported receiving information about STH and access to treatment through church channels, highlighting the influence of religious leaders in shaping health behaviors and program acceptance within these communities. Leveraging these community structures could enhance the reach and effectiveness of future interventions aimed at reducing STH prevalence and improving overall public health outcomes.

## Conclusion

Integrating these findings into targeted public health interventions is critical to achieving WHO’s soil-transmitted helminthiasis (STH) elimination goals. The evidence from this study highlights the need to revise the treatment strategy to include adolescents comprehensively in all Mass Drug Administration (MDA) interventions in Anambra State, Nigeria. By focusing on comprehensive strategies that encompass targeted health education, improved sanitation infrastructure, and inclusive treatment programs, Anaocha LGA and similar regions can significantly reduce STH prevalence and enhance overall public health outcomes.

Addressing these gaps requires specific actions, such as implementing health education campaigns in schools, upgrading sanitation facilities to ensure access to safe and functional toilets, and ensuring consistent and timely deworming treatments for all demographic groups. These efforts will not only reduce the transmission of STH but also improve the health, education, and future opportunities of adolescents, especially girls, who are disproportionately affected by these infections. By adopting a holistic approach that integrates these strategies, Anambra State can make significant strides toward meeting global health targets and serve as a model for other regions facing similar public health challenges.

### Limitations

Intervention programs should be conducted when all demographic groups can participate, to avoid exclusion. Specifically, senior secondary class 3 (SS3) students were excluded due to their participation in the ongoing West African Senior Secondary Examinations (WASSCE) at the time of the study. This exclusion may impact the generalizability of the findings across the entire adolescent population, potentially leading to an underestimation of STH prevalence among older students.

Additionally, there is a potential for underreporting of STH infections due to the reliance on stool sample analysis alone. While stool sample analysis is a standard method for detecting STH infections, it may not always capture intermittent or light infections, which could affect the reported prevalence rates. Future research could benefit from incorporating more sensitive diagnostic methods, such as molecular techniques, or repeated sampling to better capture the true prevalence and dynamics of STH infections in the study population.

Another limitation is that the study focused on the availability of toilet facilities rather than their utilization. While we recorded the availability and conditions of the toilet facilities, we did not assess how frequently they were used. Understanding effective usage is crucial for evaluating its impact on STH transmission. Future research should examine both the frequency and conditions of toilet facilities utilization to provide a more comprehensive understanding of how sanitation practices influence STH infection rates.

## Supporting information

S1 File The complete dataset used for the risk factor assessment in the study "soil-transmitted helminthiasis among Adolescents in Anaocha Local Government Area, Anambra State, Nigeria: Insights and recommendations for effective control. **"** This dataset includes quantitative data on socio-demographic characteristics, hygiene practices, environmental conditions, and other relevant risk factors associated with soil-transmitted helminthiasis. The data were collected through structured questionnaires and focus group discussions and presented anonymously to ensure participant confidentiality.
(XLSX)
